# Identification of Immunodominant CD4-Restricted Epitopes Co-Located with Antibody Binding Sites in Individuals Vaccinated with ALVAC-HIV and AIDSVAX B/E

**DOI:** 10.1371/journal.pone.0115582

**Published:** 2015-02-09

**Authors:** Silvia Ratto-Kim, Mark S. de Souza, Jeffrey R. Currier, Nicos Karasavvas, John Sidney, Morgane Rolland, Anais Valencia-Micolta, Sirinan Madnote, Alessandro Sette, Sorachai Nitayaphan, Punnee Pitisuttuthum, Jaranit Kaewkungwal, Supachai Rerks-Ngarm, Robert O’Connell, Nelson Michael, Merlin L. Robb, Mary Marovich, Jerome H. Kim

**Affiliations:** 1 United States Military HIV Research Program, Walter Reed Army Institute of Research, Silver Spring, MD, 20910, United States of America; 2 United States Military HIV Research Program/United States Army Medical Component, Armed Forces Research Institute of Medical Sciences, Bangkok, 10400, Thailand; 3 La Jolla Institute for Allergy and Immunology, La Jolla, CA, 92037, United States of America; 4 Thai Component, Armed Forces Research Institute of Medical Sciences, Bangkok, 10400, Thailand; 5 Vaccine Trials Centre, Faculty of Tropical Medicine, Mahidol University, Bangkok, 10400, Thailand; 6 Centre of Excellence for Biomedical and Public Health Informatics, Faculty of Tropical Medicine, Mahidol University, Bangkok, 10400, Thailand; 7 Department of Disease Control, Ministry of Public Health, Nonthaburi, 11000, Thailand; 8 Office of AIDS Research, National Institutes of Health, Bethesda, MD, 20892, United States of America; University of Hawaii, UNITED STATES

## Abstract

We performed fine epitope mapping of the CD4+ responses in the ALVAC-HIV-AIDSVAX B/E prime-boost regimen in the Thai Phase III trial (RV144). Non-transformed Env-specific T cell lines established from RV144 vaccinees were used to determine the fine epitope mapping of the V2 and C1 responses and the HLA class II restriction. Data showed that there are two CD4+ epitopes contained within the V2 loop: one encompassing the α4β7 integrin binding site (AA179-181) and the other nested between two previously described genetic sieve signatures (AA169, AA181). There was no correlation between the frequencies of CD4+ fine epitope responses and binding antibody.

## Introduction

The modest efficacy achieved by the RV144 Thai HIV vaccine trial [1] and the subsequent discovery of correlates of protection [2] has renewed interest in HIV antibody binding, specificity and their functionality in vaccine regimens. HIV vaccines tested previously in Phase II/III trials while eliciting strong CD8+ responses to HIV proteins failed to prevent infection [3]. Canarypox-based ALVAC-HIV vaccine prime with HIV Env gp120 boosts elicited weak CD8+ responses but induced both strong binding antibodies (bAb) and proliferative capacity by peripheral blood mononuclear cells (PBMC) to HIV Env [4,5,6]. The RV144 correlates of protection study showed that IgG antibody binding to gp70V1V2 inversely correlated with infection, while IgA antibody binding to Env protein directly correlated with infection [2]. Although no CD4+ T cell correlates of protection were found in that analysis, there was a trend for reduced risk of HIV infection with increasing magnitude of cytokines produced by Env-specific mononuclear cells [2]. In addition, our group has shown that the RV144 vaccine regimen induces CD4+ T cells that are specific to the V2 region of the HIV Env. These cells are also polyfunctional as measured by intracellular cytokine staining (ICS) and have cytolytic capability [7]. It was intriguing to find that ALVAC-HIV-AIDSVAX B/E prime-boost regimen focused the CD4+ T cell response in the same region that gives rise to the IgG bAb that showed a correlation with protection [2]. Due to the paucity of cells collected during RV144 it is impossible to test further other cellular immune parameters in the case control cohort. Despite this limitation, we attempted to further characterize the V2 specific CD4 T cell responses using the non-transformed Env-specific CD4+ T cell lines to determine the fine epitope mapping of the responses to the V2 and C1 region and their class II restriction.

## Materials and Methods

### PBMC samples

PBMC samples were obtained from the RV144 trial [1] and were selected as described in our previous publication [7]. Briefly, PBMC from 50 RV144 trial participants (40 vaccinees and 10 placebo recipients) collected at 6 months post completion of immunization (V9) were used to establish gp120 specific T cell lines. Thirty T cell lines were generated but only 14 demonstrated a specific response to the gp120A244 and were further expanded to increase cell number. Expanded T cell lines were frozen in aliquots of 5 million cells prior to use in the study. PBMC from placebo recipients did not yield any viable T cell lines. In addition, the corresponding plasma collected at 6 months post final vaccination were tested for bAb to gp120 C1 and V2 regions. The RV144 trial is registered at www.ClinicalTrials.gov, NCT00223080 and was reviewed and approved by many ethical committees as described in the main clinical paper RV144 [1].

### Antigens and Peptides

The HIV CRF01_AE derived A244 gp120 used for the establishment of the T cell lines was kindly provided by Marc Gurwith (GSID) and was identical to that contained in the AIDSVAX B/E vaccine. The 138 peptide set of 15–18 aa overlapping by 10–12 aa spanning CRF01_AE isolate CM235 Env protein was purchased from JPT Peptide Technologies (JPT, Berlin Germany) as described previously [7]. Truncation peptides and peptides containing mutated sequences corresponding to peptide 32 (p32) were synthesized by standard solid phase methods on an Applied Biosystems 433 Synthesizer using Fmoc chemistry (Foster City, CA), as described previously [8]. Truncation peptides corresponding to p31 and p16 were purchased from (JPT Peptide Technologies) purity was 90% or greater.

### B-Lymphoplastoid cell lines (B-LCL) and non-transformed CD4+ Env specific T cells

Generation of B-LCL and non-transformed CD4+ Env specific T cells was previously described [7]. Briefly, cryopreserved specific T-cell lines were thawed, washed and re-suspended in RPMI1640 with 10% NHS at 1x10^6^ cells/ml and then co-incubated in the presence of 5x10^5^ autologous B-LCL in the presence or absence of single peptides (1 μg/ml) for 4 hrs for ICS assays or 24 hrs for ELISpot. Assays were performed as described previously [7,9]. A positive IFN-γ response for the ELISpot and ICS assay was defined as greater then 3 times background (B-LCL and T cells with no peptide).

### Antibody binding assay

Peptide ELISA was performed using U-bottom 2HB plates or streptavidin coated plates coated with 1 μg/mL of cyclic V2 or C1 peptide, respectively, in D-PBS at 4°C overnight as described previously [10].

### HLA typing

Samples were HLA Sequenced Based Type at the University of Oklahoma Health Science, a CLIA/ASHI accredited laboratory, using in-house PCR and sequencing methodologies. The entirety of exon 2 was DNA sequenced for all class II loci with additional exons sequenced for DQB1 (exon 3), DQA1 (exon 3), and DPB1 (exons 3 & 4). DNA sequence analysis and HLA allele assignment were performed with the software Assign-SBT v3.5.1 (Conexio Genomics). The HLA database for allele assignment was updated with IMGT release 3.0.0 May 5th 2010. Any ambiguous types that remained following DNA Sequence Based Typing were resolved to 4-digits using the PEL-FREEZ UNITRAY SSP, Life Technologies.

### HLA algorithm

HLA class II binding predictions were performed using the IEDB’s (www.iedb.org) Analysis Resource consensus algorithm [11]. Peptides with consensus percentile scores <20 (in general corresponding to an affinity > 1000 nM) were classified as intermediate binders, and those with percentile scores <10 (~100 nM) were defined as high affinity binders.

## Results

### Fine Epitope mapping of Env-specific CD4+ T cell lines

**Figure 1:**
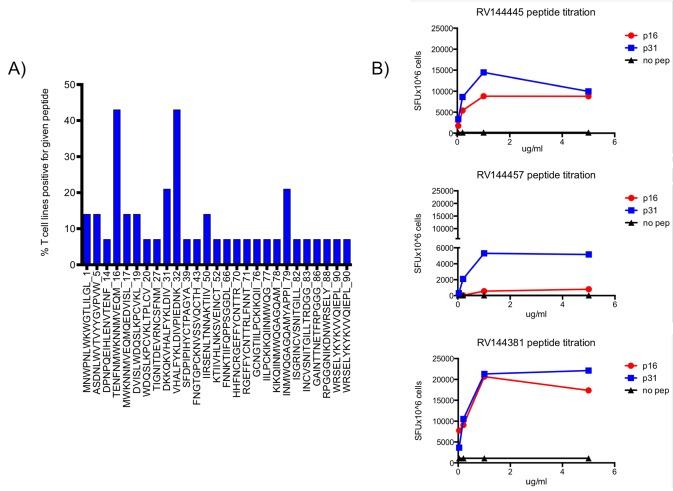
Envelope specific CD4+ T cell responses in RV144 vaccine volunteers. A) Graphical representation of the percentage of gp120A244 specific CD4+ T cell lines (N = 14) generated from RV144 volunteers that recognize a given peptide. The peptide sequence is showed on the X axis, number at the C-terminus is the sequential peptide number in the pool. B) Titration of the gp120A244 specific CD4+ T cell lines peptide responses. Three T cell lines responding to pep16 and pep31 are shown. Responses were measured in a modified ELISpot assay. Peptide concentration is shown on the X axes.

Env-specific CD4+ T cell lines were established from 14 volunteers enrolled in RV144, tested with a series of pooled peptides in a matrix format and subsequently tested with the defined single peptide for confirmation. Fig. 1A and S1 Table shows the frequency of recognition of the peptides for the 14 T cell lines: there are 4 areas where 3 or more CD4 T cell lines recognize the same epitope: one in C1 (p16) 2 in V2 (p31 and p32) and 1 at the beginning of C4 (p79). In general the median number of peptide recognized by a given T cell line was 3 and the range was [1–6].

The Env-specific CD4+ T cell lines were clearly polyclonal and each clone present in the culture responded to a given peptide in a dose-dependent manner (Fig. 1B and S2 Table).

**Figure 2:**
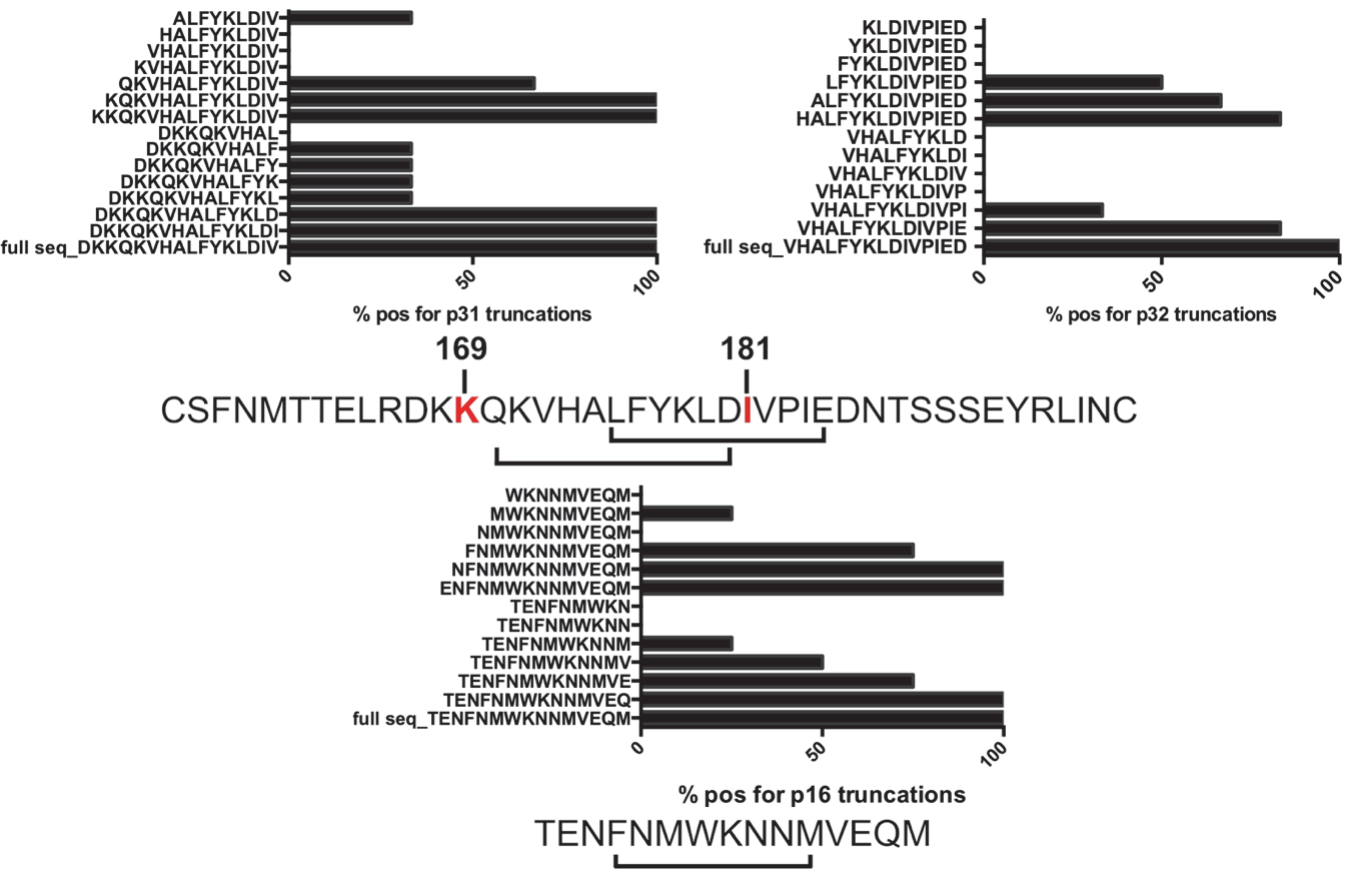
Env-specific CD4+ T cell lines that showed positive responses to either p16, p31 or p32, were tested in a modified ELISpot against a panel of truncated peptides based on the full sequence of p16 (gp120 C1 region), p31 and p32 (gp120 V2 region). Addition of Q (aa 170) increase % positive to about 60% and 100% response is reached when K (aa169) is added. Position 180 (D) is also critical as the number of responders increased from about 30% to 100%. This position is extremely conserved and forms the α4β7-binding motif. Amino acid at position 176 (F) is important for antibody binding. HAL aa sequence is within a highly conserved region of the V2 loop. Sequences below the graph depict where the mapped epitope lies. The amino acids within boxes were sieve signatures described by Rolland et al. [12].

We defined the fine epitope specificity using a series of truncated peptides that span the sequences of the V2 peptides and C1 peptide. Reagents to study the fine epitope specificity for p79 were not available at the time of the study. Fig. 2 and S1 Table shows the frequency of the T cell lines with V2 and C1 specificity. These experiments identified two epitopes in V2 overlapping by 6 aa but not cross-reactive as individuals that recognized peptide 31 (p31) did not recognize peptide 32 (p32) despite 11 aa of overlap. A peptide truncation set spanning peptide 16 (p16) revealed a possible shorter epitope that was not contained in the adjacent overlapping peptide 17. Interestingly, the CD4+ T cell epitopes defined in the V2 region contained or fell in between the V2 K169Q and I181L sieve mutations in RV144 vaccine recipients who subsequently became HIV infected [12] (Fig. 2 and S1 Table).

**Figure 3:**
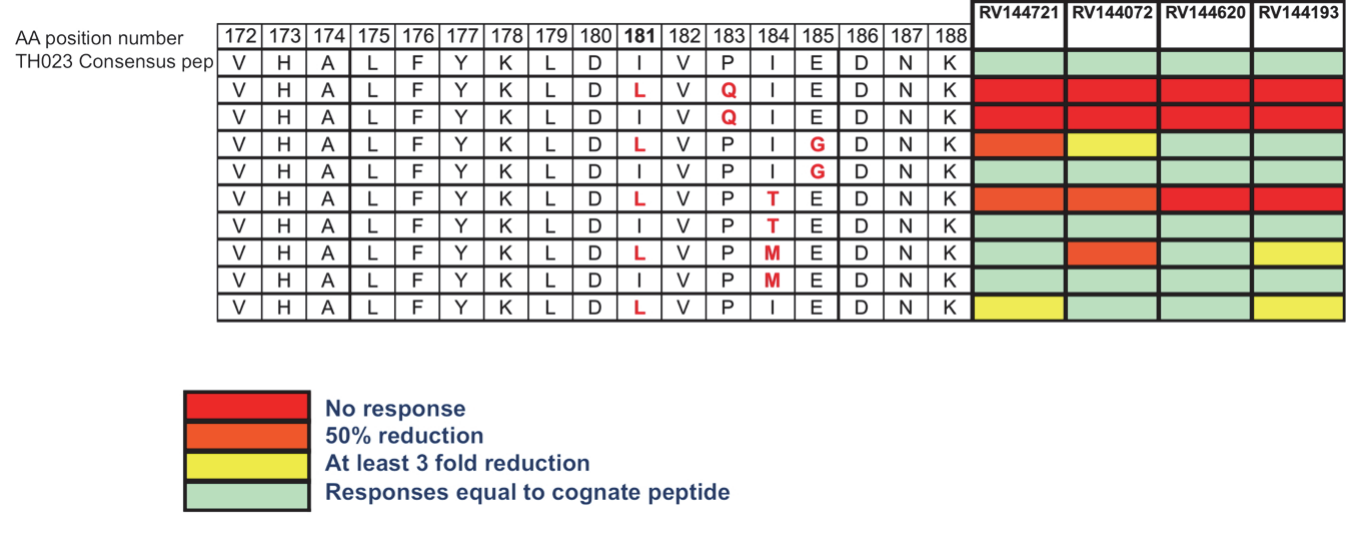
Peptide 32 is shown in comparison to other synthesized peptides containing sieve mutations at position 181 and other mutations (at positions 183–185) that were observed in the viruses found in vaccinee breakthrough infections. The heat map on the right represents the IFN-γ fold reduction response of the 4 gp120A244 specific CD4+ T cell lines to the altered peptides relative to the consensus sequence. The aa mutation P183Q, was similarly expressed between the the two arms with breakthrough HIV infections. In particular, in the 44 vaccinees, 16 were P (36%), 22 Q (50%), and 5 other residues (E,K,N,S) were also most common. For the 66 Placebo recipients, 28 were P (42%), 31Q (47%), and 7 were other residues (E,K,R,S).

In an attempt to understand if this mutation had any effect on the CD4+ recognition of the epitope a series of peptides were synthesized that contained the aa mutation at position 181 (I to L mutation) and other mutations that although not significantly associated with the vaccine treatment, were also common in the HIV sequences from the breakthrough infections in RV144. The peptide that contained the I181L mutation on its own was recognized in an ICS assay as well as the original peptide (Fig. 3 and S3 Table). The aa mutation P183Q, that abolished peptide recognition was not differentially expressed between vaccine and placebo breakthrough viruses.

### MHC class II restriction analysis

**Figure 4:**
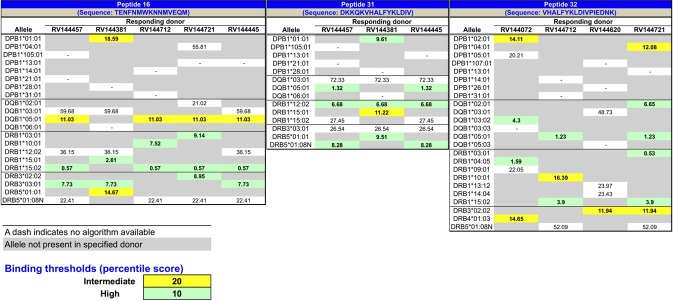
The figure shows the alleles present in any of the donors that responded to the specific epitope and the HLA binding prediction analysis. Accordingly, where possible, these algorithms were deployed to predict the binding of each recognized envelope peptide to the HLA class II alleles present in the corresponding responding individuals. Analysis of the resulting pattern of predicted binding was used in turn to infer HLA restriction.

MHC class II restriction of the T cell lines was performed using a HLA class II restriction prediction analysis. The analysis is summarized in Fig. 4 and shows the parsed data by peptide recognition. DRB1*15:02 is strongly suggested to be a restricting allele for p16. Four of the five donors responding to the peptide carry this allele, which is predicted to bind with high affinity. The donor that did not have DRB1*15:02 had15:01, which has a binding characteristic similar to 15:02. However, it is possible that DRB3 or DQB1*05:01 could play a role too. All three donors responding to p31 expressed DRB1*12:02. As p31 is predicted to bind this allele with high affinity, suggesting that DRB1*12:02 is at least one restriction element for p31. DRB5*01 is similarly present in all three donors, and high binding is similarly predicted. A consistent pattern was more difficult to identify for p32. No allele is present in more than two of the four donors recognizing the peptide. At the same time, multiple DR and DQ alleles are predicted to bind the peptide with high affinity.

### Humoral response in the individuals from whom the CD4+ T cell lines were established

Plasma from the same volunteers was available from the same time point from which the Env-specific lines were developed. BAb assays were performed using a Cyclic V2 peptide that contains both CD4 T cell V2 epitopes and a biotinylated linear peptide identical to the C1 peptide. Of the 9 individuals that had a CD4+ T cell response to V2, 4 had also a positive bAb response. Conversely, there were 3 individuals who had a bAb response to V2 but the corresponding T cell line epitope mapping did not include a positive response to V2 (Table 1 and S4 Table). A similar pattern was seen for the bAb response to C1 and the T cell line p16 recognition.

**Table 1:** Comparison between bAb responses and CD4+ T helper responses to C1 or V2 (p31 or p32 as indicated) respectively within the same individual.

	CD4 T helper C1	bAb C1	CD4 T helper V2	bAb V2
RV144072			X (p32)	
RV144621				
RV144457	X		X (p31)	
RV144100				
RV144929			X (p32)	
RV144381	X		X (p31)	X
RV144712		X	X (p32)	
RV144453	X	X		X
RV144716	X			X
RV144620			X (p32)	X
RV144721	X		X (p32)	
RV144193			X (p32)	X
RV144445	X	X	X (p31)	X
RV144396	X			X

X: mark represents the presence in that individual of a positive response by the assay indicated. The p value for the 2x2 contingency table comparison using one tail Fisher exact was >0.05 for both comparisons (C1 Ab vs C1 CD4 responses and V2 Ab vs V2 CD4responses).

## Discussion

Initial studies of the immunogenicity of the ALVAC-HIV and AIDSVAX B/E vaccines used in RV144 showed an average proliferative response to Env of 70% and while the CD8+ responses to Env were marginal in an ICS assay (11%) the CD4+ reactivity by ICS was observed in 34% of vaccine recipients [1]. Previous work had demonstrated cytolytic CD4+ T cells specific for the V2 region of Env [7]. While a formal correlates of risk study identified two antibody-dependent correlates, there was a suggestion that CD4 responses might (albeit weakly) be associated inversely with the risk of infection [2]. Using non-transformed, Env-specific CD4+ T cell lines, we evaluated the fine specificity of CD4+ T cell responses and HLA restriction.

The epitope mapping revealed common epitopes in C1 and C4 and two immunodominant V2 epitopes. These regions have already been described in the Los Alamos database as potential CD4+ T helper epitopes [13]. Interestingly, the two distinct CD4+ T helper epitopes in V2 epitope spanned the I181L sieve mutation described by Rolland et al. and the other falls between the K169Q and I181L sieve signatures [12]. Mutation I181L did not alter CD4 T cell line recognition of the epitope, suggesting that sieving immunological pressure may have come from the V2 bAb induced by the vaccine. We did not find a correlation between anti-V2 bAb and V2 specific T cell responses. The T cell lines and bAb were identified at visit 9 (6 months post final vaccination), which is not peak immunogenicity, and bAb appear to have decreased 20-fold over time [1], therefore it could be argued that our study was not conducted at the best time point to assert a correlation. Additionally, because the T cell-lines are expanded in vitro, it is possible that V2 specific clones were lost due to culture conditions.

Reports have shown that immunodominant epitopes may be shared between T cells and antibody but proof of a causal relationship has been difficult to obtain. A murine model seems to show a functional link between T and B cells that recognize overlapping epitopes [14]. However, in RV144, the post-vaccination predominance of V2 CD4 T cell responses is not found after vaccine recipients become infected, as individuals with breakthrough infections did not have detectable cellular responses to V2 by ELISpot compare with 50% in the vaccinated non-infected individuals (M. de Souza unpublished observations). It is possible to argue that despite their cytolytic phenotype, V2 specific CD4+ T helper cells induced by the vaccine may be recruited to the site of infection and subsequently become infected and are eliminated, as suggested in HIV infection [15].

Although the frequencies of HLA class II alleles in Thailand have been reported [16], specific reagents to allow in vitro confirmation of peptide binding to the most prevalent alleles are still lacking. For this reason, HLA class II restriction analyses were performed using consensus binding prediction algorithms available through the IEDB’s Analysis Resource web site. However, because predictions could not be made for all alleles, and because the binding patterns could not be confirmed with *in vitro* assays, the patterns of restriction suggested will require future studies to provide further elucidation and confirmation. Nonetheless, we can remark that the two most promising allele/peptide associations were for DRB1*12:02 for p31 and DRB1*15:02 for p16, corresponding to two alleles found with high frequency in Thailand [16].

In conclusion, we have shown that fine epitope mapping of the CD4+ T cell responses in RV144 individuals identifies epitopes in a highly immunogenic portion of the Env protein. In particular the two epitopes identified in the V2 regions span the antibody-binding site that correlated with reduced risk of infection in RV144 [2]. In addition, the possible HLA class II restrictions were identified. More studies are warranted to better understand the function of the Env specific CD4+ T cell response in HIV vaccine studies.

## Supporting Information

S1 Table Analysis of peptide positivity by ELISpot. Only peptide sequences that were scored positive are indicated in the first column of each table. Each tab shows the peptides set used and their corresponding responses.
(XLSX)

S2 Table ELISpot data used in the peptide titrations graphs. (PZF)

S3 Table Percentages of intracellular cytokine staining produced by CD4+ T cell lines in response to a set of peptides that include sieve mutations or mutations that were observed in the breakthrough infection from the RV144 trial. The first two tabs represent two experiments using in part the same peptides. The third tab show the summary of the analysis.
(XLSX)

S4 Table Antibody binding measurements. The Table shows duplicate readings of absorbance measured at 450 nm for the same volunteers that envelope specific CD4+ T cell lines were established.
(XLSX)
